# SPECT and PET myocardial perfusion imaging in Austria, Germany, and Switzerland results of the 2nd joint survey 2024

**DOI:** 10.1007/s00259-026-07790-w

**Published:** 2026-02-20

**Authors:** Oliver Lindner, J. Bucerius, T. Derlin, W. Burchert, R. R. Buechel

**Affiliations:** 1 Institute for Radiology, Nuclear Medicine, and Molecular Imaging, Heart and Diabetes Center NRW, Ruhr University Bochum, Bad Oeynhausen, Germany; 2 Department of Radiology, Division of Nuclear Medicine, Medical University of Graz, Graz, Austria; 3 Department of Nuclear Medicine, Cardiac Imaging, University Hospital Zurich, Zurich, Switzerland

**Keywords:** Myocardial perfusion scintigraphy, Utilisation review, Utilisation statistics, Trends

## Abstract

**Purpose:**

We herein present the results of the second survey on SPECT and PET myocardial perfusion imaging (MPI) in Austria, Germany, and Switzerland in 2024.

**Methods:**

A questionnaire was sent to facilities practicing nuclear medicine.

**Results:**

Data from 12 Austrian (10,689 SPECT), 198 German (128,707 SPECT), and 16 Swiss departments (11,593 MPI (2,911 SPECT; 8,682 PET)) were analysed. In Austria and Germany, the PET MPI numbers were negligible and not considered. In Austria 50%, in Germany 69%, and in Switzerland 69% of the facilities reported stable or increasing numbers of examinations compared to the 2021 survey. Ambulatory care cardiologists represented the major referral group (46-71%). Most stress tests were performed pharmacologically (57-96%). In SPECT imaging, the one-day protocol was predominant in Switzerland (77%), while the two-day protocol was used in Austria (41%) and Germany (49%). The primary camera systems used were hybrid SPECT-CT systems in Austria (75%) and Switzerland (100%), and SPECT cameras (without CT) in Germany (56%). Attenuation correction was regularly performed in Switzerland (100%), followed by Austria (74%), and Germany (35%). Both gated SPECT and perfusion scoring were frequently applied (gated SPECT 82-99%; perfusion scoring 82-88%).

**Conclusions:**

This second joint survey for 2024 confirms positive trends in MPI imaging in all three countries, albeit with differences. The results document a high level of guideline conformity. The situation in Switzerland is exceptional due to the widespread use of PET-MPI. Switzerland also leads in terms of camera equipment and attenuation correction followed by Austria and Germany.

## Introduction

We herein present the results of the second joint survey on SPECT and PET myocardial perfusion imaging (MPI) in 2024 in Austria, Germany, and Switzerland. The results of the first joint survey of the year 2021 have been published in this journal [1]. The three-year period is based on the cycle of the German survey, which has been in place since 2009. Although the German data have already been published [2], it is listed again for better comparability, but without differentiation by type of institution (i.e., practices, hospitals, university hospitals). The data is exceptionally important for guiding future development in cardiac imaging on a rational basis.

## Methods

In Austria, departments performing MPI were identified with the help of the Austrian Society of Nuclear Medicine and Theranostics (OGNT) supplemented by addresses from the German hospital address book [3].

In Germany, the updated database from the 2021 survey was used to contact departments and physicians practicing nuclear medicine.

In Switzerland, departments performing MPI were identified with the help of the Swiss Society of Nuclear Medicine (SGNM). All departments were directly contacted through the Swiss Society of Nuclear Medicine.

A one-page questionnaire, accompanied by a cover letter, was sent via fax or email in the first quarter of 2025. If no feedback was received, a reminder was sent four weeks later, followed by a second reminder four to six weeks after that. The survey closed at the beginning of May 2025. The questions to be answered in all countries were:

- number of patients examined,
- number of stress and rest procedures,
- number of different types of stress tests,
- number of patients by study protocol,
- percentage of patients examined with gated SPECT,
- percentage of patients examined with attenuation correction (AC),
- usage of semiquantitative scoring *(categories: never*,* always*,* intermediate (= between “never” and “always”))*,
- type of gamma camera (multiple entries possible),
- use of Tl-201 during the generator shortage in the fall of 2024.
- percent referrals from cardiologists, primary care physicians, hospital physicians, and others,
- changes in referral *(categories: no change*,* unchanged*,* more*,* unknown)* and in case of a decline, potential cause or causes *(stress-echocardiography*,* cardiac CT*,* cardiac MRI*,* invasive coronary angiography; multiple entries possible)*,
- number of bone scans for diagnosing cardiac amyloidosis, and estimated positive rate (this data has already been published [4]).

A copy of the questionnaire is added in the supplement of [4].

In Switzerland, additional figures were requested on: (1) the number of cardiac PET scans, (2) the PET radiopharmaceuticals used, and (3) the number of PET scans in assessment for cardiac inflammation and endocarditis.

To verify the representativeness of the survey and to reliably estimate the total of SPECT MPI numbers, the German survey figures were related to the official data delivered by the National Association of Statutory Health Insurance Physicians (NASHIP) (Kassenärztliche Bundesvereinigung (KBV; www.kbv.de)) as described [5].

For Austria, official SPECT MPI numbers were delivered by the „Gesundheit Österreich GmbH“ (GÖG; www.goeg.at), the national health research and planning institute and central agency for health promotion under the supervision of the Federal Ministry of Social Affairs, Health, Long-Term Care and Consumer Protection. The total number of examinations in Austria was calculated based on inpatient, hospital outpatient, and non-hospital examinations. At the time of writing this manuscript, the 2024 figure of the outpatients, which was about 40% of the total number in the past years, was unavailable and thus estimated based on the average change over the last five years.

In Switzerland, no official data was available.

## Results

### MPI numbers and regional distribution

Table 1 lists the number of MPI patients recorded in the surveys. In both Austria and Germany, nearly no PET MPI was performed. Therefore, no data is listed.

In Austria, 40 facilities practicing nuclear medicine were identified. Feedback was provided by 12 (30%). In Switzerland, a total of 21 facilities known to provide nuclear cardiac imaging services were invited of which 16 (76%) took part in the present survey.

In Germany, questionnaires were sent to 628 nuclear medicine and radiology facilities or individuals that were performing some form of nuclear medicine. Feedback was provided in 222 cases. Of these, 24 reported that no MPS were performed in 2024 or at all.

**Table 1:** MPI procedures in Austria, Germany, and Switzerland in 2024

	Austria	Germany	Switzerland		
	SPECT	SPECT	SPECT	PET	Combined
Number of facilities	12 [14]	198 [218]	15 [16]	9 (9 Rb; 2 NH_3_) [6 (5 Rb; 2 NH_3_)]	16 [16]
No. of patients in the survey	10,689 [10,710]	128,707 [133,057]	2911 [6879]	8682 [4722] Rb 6733 [3041] NH_3_ 1949 [1681]	11,593 [11,601]
Total patients*	29,289 [27,030]	242,843 [246,402]	-	-	-
% total patients	36 [39]	53 [54]	-	-	-
MPI/100,000 pop*	272 [292]	281 [303]	-	-	-
Mean/facility	891 [765]	650 [610]	194 [430]	964 [787]	724 [725]
Median/facility	360 [517]	396 [392]	63,5 [389]	796 [842,5]	568 [439]
Range/facility	182–2314 [170–1699]	2-6500 [1-5613]	1-766 [4-1121]	380–1949 [156–1272]	45-1964 [4-2056]

In square brackets 2021 data

Austrian population: 7.96 million, German population: 84.67 million, Swiss population: 8.96 million

* Austria: data according to GÖG (Gesundheit Österreich GmbH; www.goeg.at); Germany: estimate based on the statistics of NASHIP (National Association of Statutory Health Insurance Physicians)

**Fig. 1:**
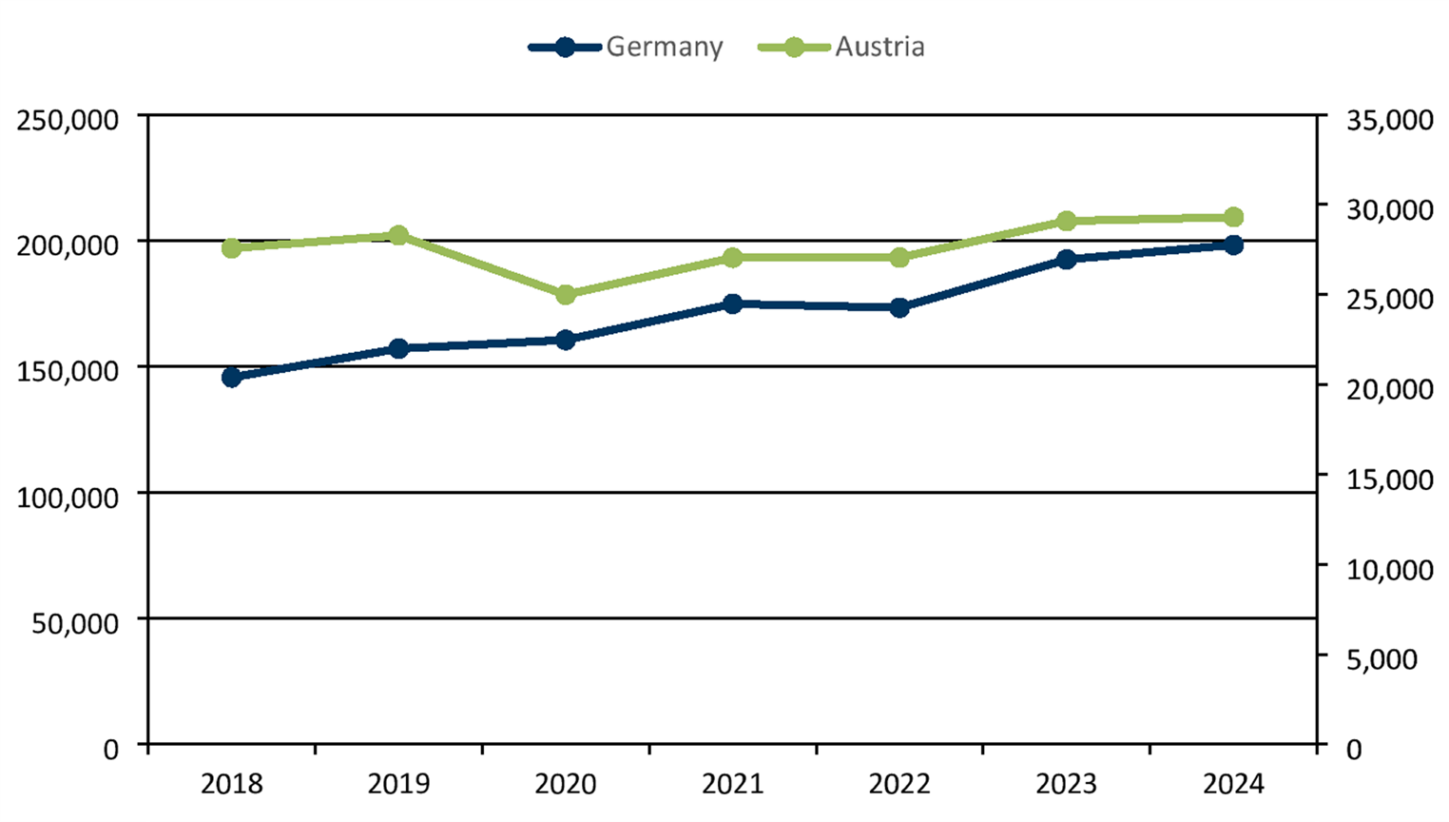
Official SPECT MPI numbers in Austria and Germany from 2018 until 2024. *Left hand scale*: Germany; *right hand scale*: Austria

Figure 1 shows the time course of the official data in Austria and Germany from 2018. Data from Switzerland are not available. For Austria, the code DA010 (myocardial scintigraphy) of the Austrian benefit catalogue, and for Germany, the fee schedule item 17,330 (stress SPECT MPI) of the German Uniform Assessment Standard (EBM, Einheitlicher Bewertungsmaßstab) is listed.

### Changes in MPI referral from 2021 to 2024 and competitive methods

**Fig. 2:**
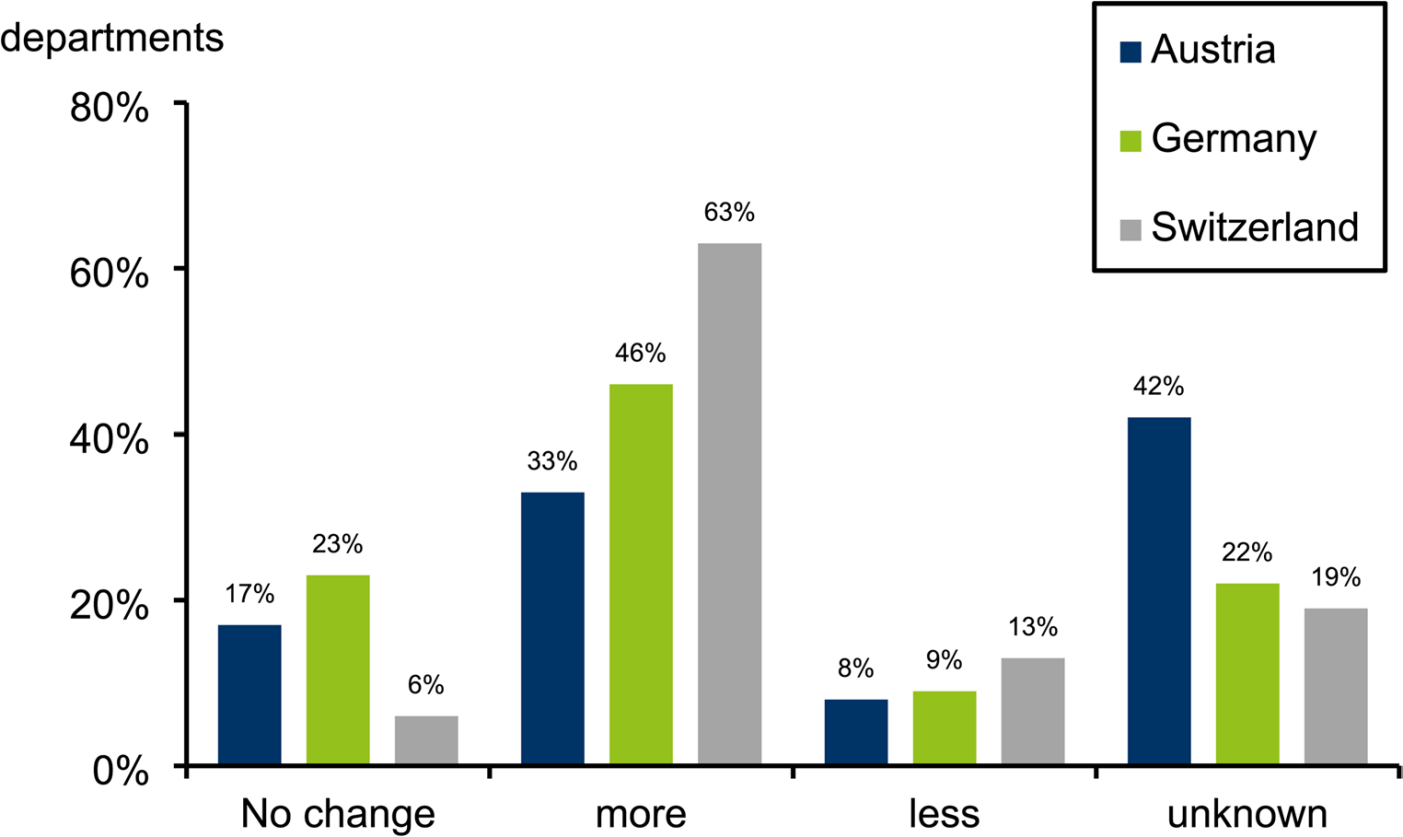
Changes in MPI referral from 2021 to 2024 in the departments by country

The changes in MPI referral are depicted in Fig. 2. In the 2024 survey, we asked about changes in referrals since the last 2021 survey 2021 for the first time in all countries.

In Switzerland, MRI was stated in all cases as the reason for the decline, in Austria both CT coronary angiography and MRI, and in Germany MRI, invasive coronary angiography or a combination of alternative imaging methods.

The mean value of those with no change, more, less, or unknown in Austria was 1153, 874, 919, 288, in Germany 787, 897, 353, 319, and in Switzerland 1293, 796, 393, 576.5.

### Referrer structure

**Fig. 3:**
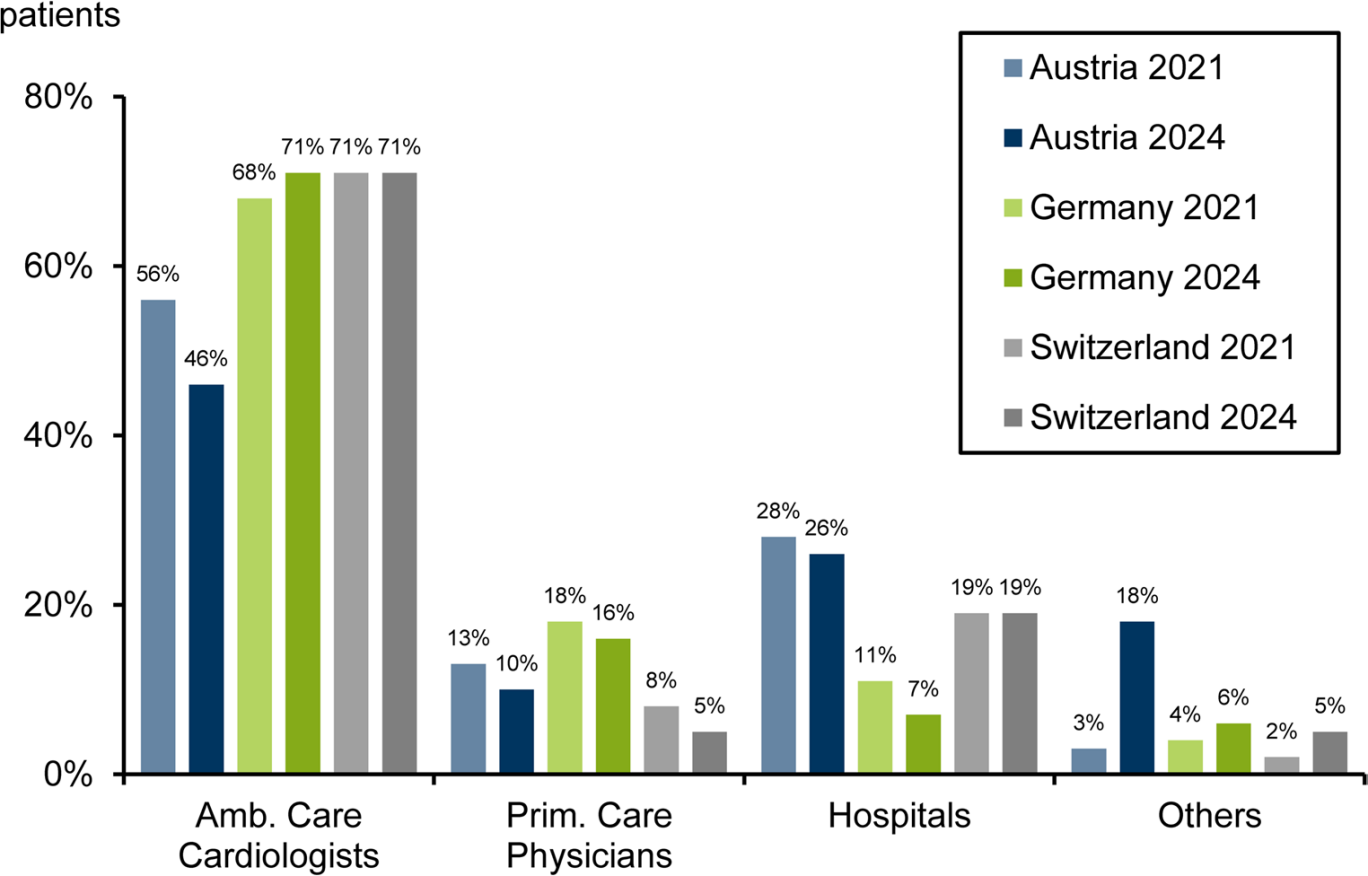
Referrer structure by country in 2021 and 2024

Figure 3 shows the referrer structure to SPECT and PET MPI. The pattern was roughly similar in all countries. Ambulatory care cardiologists represented the major referrer group (> 40%). In Austria, the hospital proportion was highest among the three countries, and a shift in referrals from cardiologists to other specialists (mostly internists) was observed.

### Stress tests

**Fig. 4:**
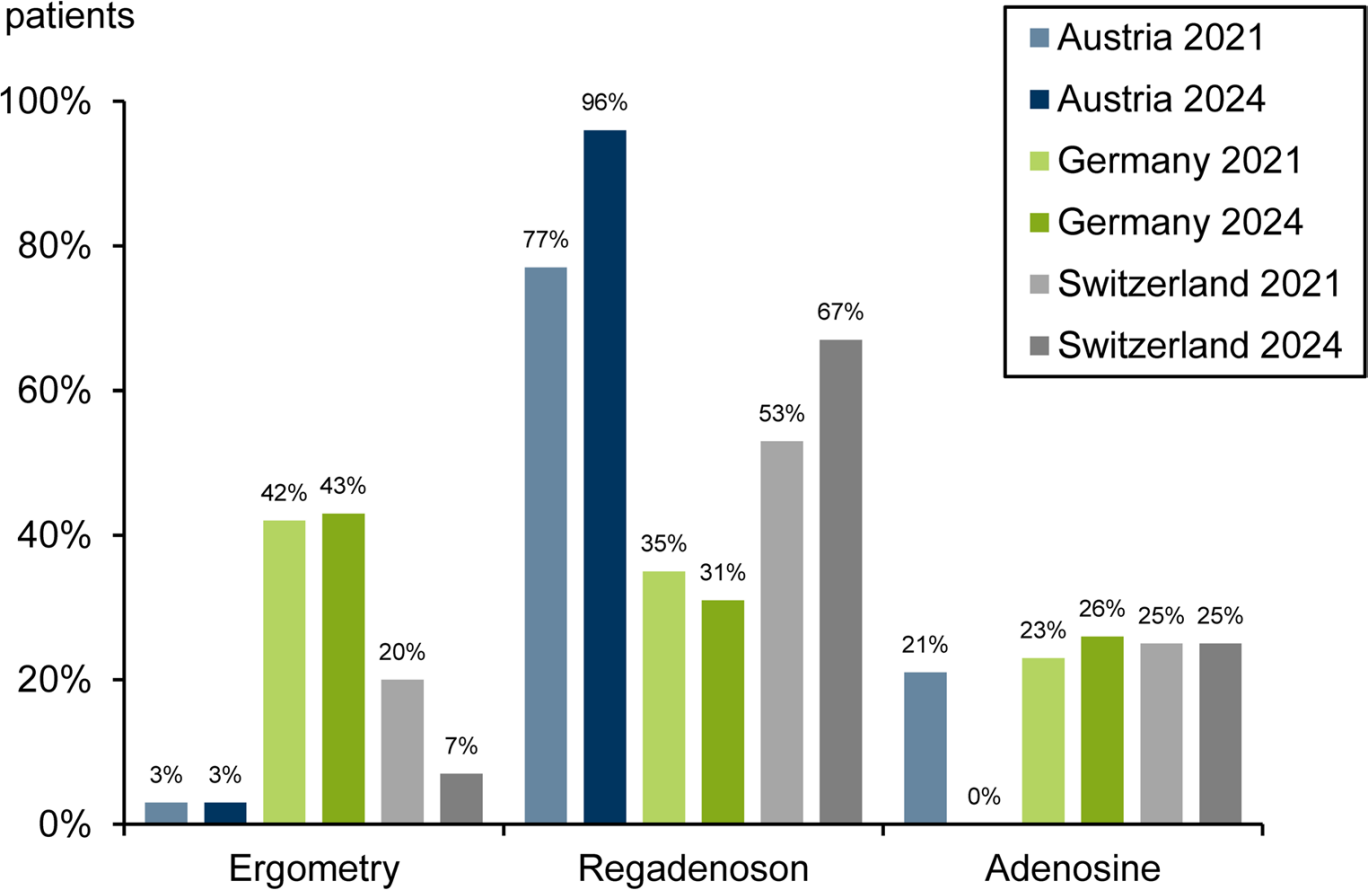
Stress tests by country in 2021 and 2024. Dobutamine: Austria 0% [0%], Germany 0.1% [0.1%], Switzerland 2% [1%]

The use of the various stress modalities and agents is illustrated in Fig. 4.

Stress tests were predominantly performed pharmacologically in all countries. Of the three countries, Germany still had the highest rate of ergometry. However, it played almost no role at all in Austria and Switzerland. Regadenoson was the leading pharmacological stress agent, experiencing strong growth in Austria and Switzerland. Adenosine was no longer used in Austria by 2024. As expected, the proportion of the second-choice stressor dobutamine was very low (≤ 1%).

### Protocols

**Fig. 5:**
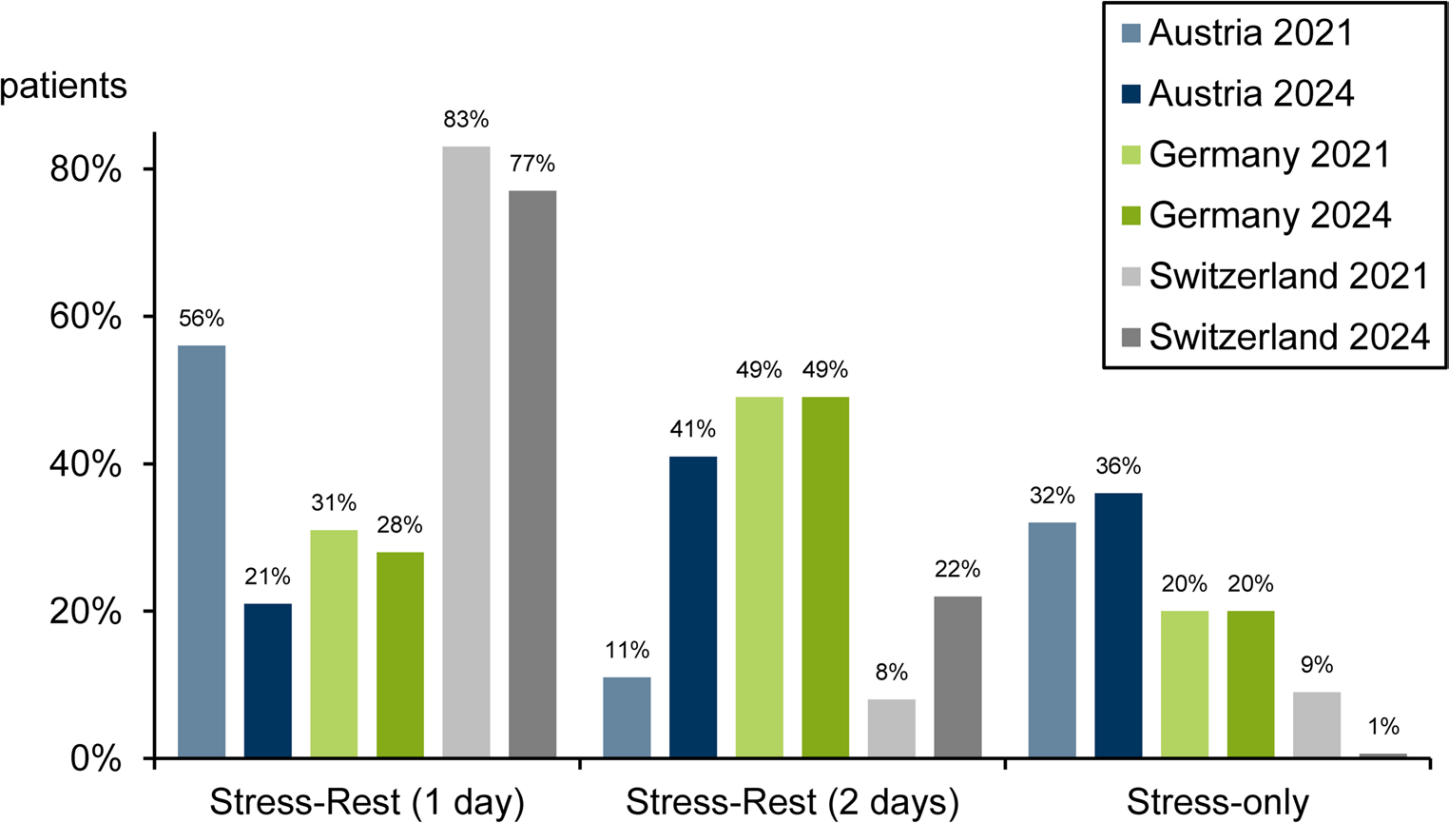
SPECT protocols by country in 2021 and 2024. The difference to 100% represents the small proportion of rest-only protocols not shown in this figure

The use of the different SPECT study protocols is shown in Fig. 5.

In Germany, the use of protocols remained nearly unchanged, while in Austria, there was a shift from 1-day protocols to 2-day protocols, and in Switzerland, a decline in stress-only protocols to 1% was accompanied by a corresponding increase in 2-day protocols.

Stress-only imaging was most frequently performed in Austria, with less than 10% in Switzerland and Germany in between.

Rest-only protocols were used very rarely (< 1%).

During the generator shortage in autumn 2024, 13 German departments temporarily used Tl-201 in 953 patients, while 3 Austrian departments used it in 55 patients; however, none of the Swiss departments did so.

### Camera systems

**Fig. 6:**
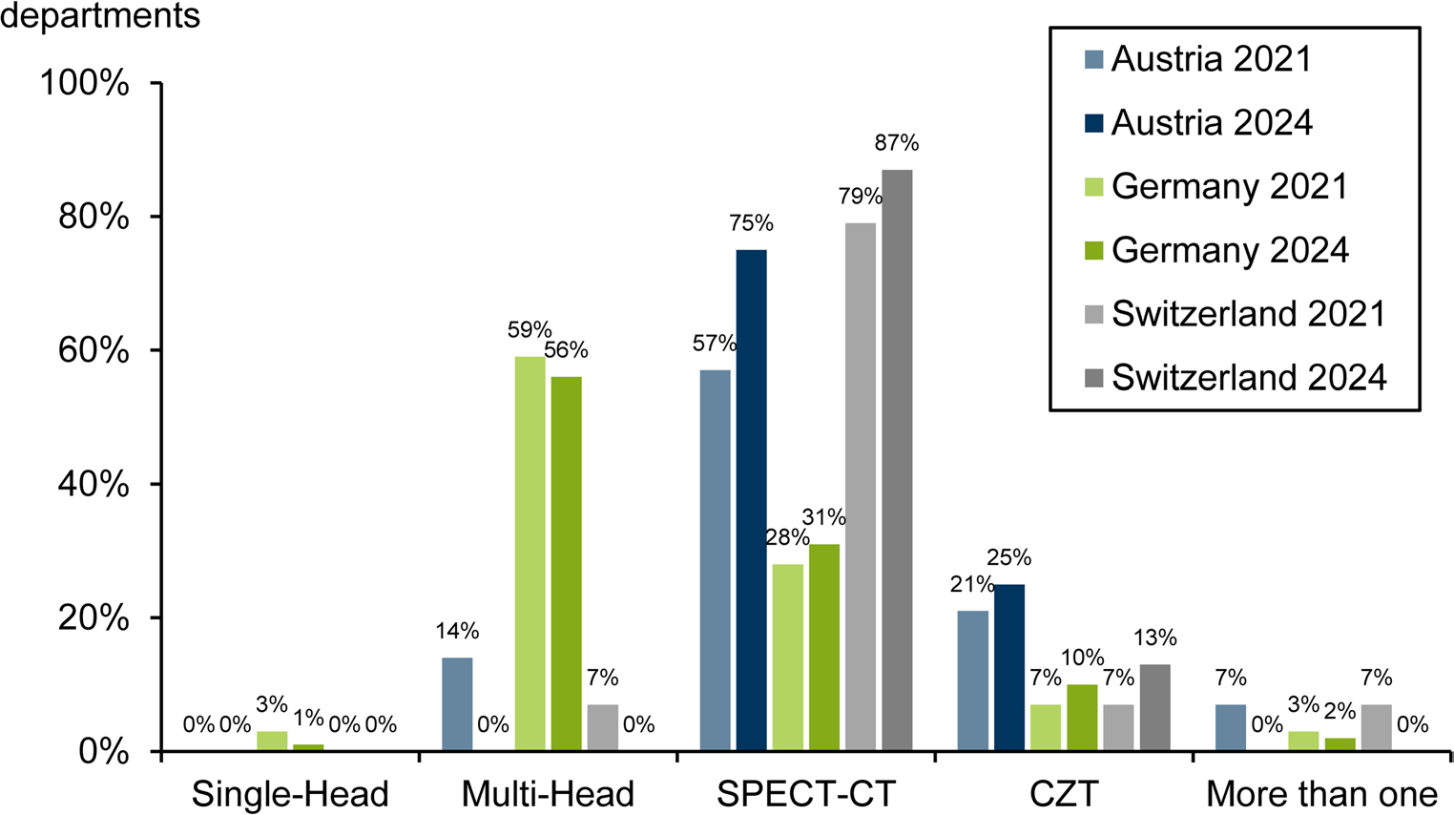
Camera systems by country in 2021 and 2024

The camera systems used for SPECT MPI are depicted in Fig. 6.

In the 2024 survey, dedicated cardiac cameras and CZT cameras were summarised under the heading ‘CZT’. Correspondingly, the 2021 data were adjusted in the figure.

Single-head cameras were still in use in German facilities, albeit to a minimal extent. The leading camera system in Austria and Switzerland was SPECT-CT, with an increase in both countries. Switzerland had the highest proportion. In Germany, these systems were used to a considerably lesser extent for MPI and showed a considerably smaller increase.

Multi-head cameras remained the main system for MPI in Germany. In Austria and Switzerland, they were no longer used.

CZT cameras recorded a slight increase in all countries. Austria continued to account for the largest share.

### Attenuation correction

The number of patients examined with attenuation correction systems corresponds to the number of SPECT-CTs in each country. In Switzerland, all patients underwent examination with attenuation correction, indicating the need for additional equipment in CZT systems, such as AI-based methods for attenuation correction, or the use of a standalone CT for obtaining attenuation maps (Table 2).

In square brackets 2021 data.

### ECG-gated SPECT and scoring

Data are listed in Tables 3 and 4. All countries showed high usage, with near-perfect values in Switzerland. Austria showed a considerable increase in ECG-gated SPECT of the rest study.

**Table 2:** Attenuation correction in MPI by country

	Austria	Germany	Switzerland
% patients studied with attenuation correction	74 [43]	35 [33]	100 [72]

In square brackets 2021 data

**Table 3:** MPI performed as ECG-gated SPECT by country

	Austria	Germany	Switzerland
Gated stress	95% [87%]	95% [89%]	99% [99%]
Gated rest	95% [61%]	93% [88%]	99% [99%]
Gated both	95% [60%]	92% [87%]	99% [99%]

Data represent percentages of MPI performed as ECG-gated SPECT. In square brackets 2021 data

Perfusion scoring, in general, experienced a positive trend. Standard use of scoring has now reached a similar level in all countries, with Switzerland ahead.

**Table 4:** Utilisation of perfusion scores by country

	Austria	Germany	Switzerland
Always	82% [64%]	84% [72%]	88% [60%]
Intermediate	8% [22%]	12% [15%]	6% [33%]
Never	10% [14%]	4% [13%]	6% [7%]

Data represent percentages of facilities. In square brackets 2021 data

### Imaging for cardiac inflammation and endocarditis

Exclusively in Switzerland, the number of cardiac PET scans for assessing cardiac inflammation and endocarditis was also requested.

In total, 1094 scans were performed for cardiac inflammation (primarily for evaluation of suspected cardiac sarcoidosis or therapy monitoring in case of known cardiac involvement), and 171 in the setting of suspected endocarditis.

The results will not be discussed further here but will serve as a basis for future surveys to assess the trend of cardiac PET imaging beyond ischemic heart disease.

## Discussion

This paper presents the results of the second joint survey of the year 2024 on SPECT and PET MPI in Austria, Germany and Switzerland. The first joint survey covered the year 2021 [1].

Based on official examination numbers, this survey included 36% of all patient examinations in Austria and 53% in Germany. As already mentioned, it was not possible to estimate the figures for Switzerland due to the unavailability of official data.

It cannot be ruled out that the results may be biased due to the non-responding centres, the exact number of which is unknown.

Nevertheless, the results of increasing MPI figures, which are officially confirmed, and the performance data derived from them allow for a reliable assessment of the development presented at least of Austria and Germany. Considering that the numbers of examinations and the total population in Austria and Switzerland are quite similar, the Swiss data can also be considered representative.

For a better understanding of the results of the different use of cardiac PET MPI, it is necessary to consider that the countries have different framework conditions in their healthcare systems. There is reimbursement for cardiac PET MPI in Switzerland, which is not the case in Austria or Germany. In Austria, nuclear medicine is centralized within hospitals, where PET is used almost exclusively for oncological indications. In Germany, MPI is predominantly (> 70%) performed in outpatient practices [2]. As in Austria, PET is mainly used for oncology.

These facts help to explain the striking differences in the use of PET MPI in Switzerland compared to Austria and Germany. In the latter countries, cardiac PET is only performed in a small number of facilities. Exact numbers are not available, but they are undoubtedly negligible.

An encouraging development is the continuing positive trend of SPECT MPI numbers in Austria and Germany in recent years. As shown in Fig. 1, except for the first year of the pandemic in 2020, the development is similar in both countries.

An analysis of the overall figures reveals that there is little difference between Germany and Austria in terms of the number of examinations per 100,000 inhabitants. It should be noted that the official Austrian figure for 2024 is based in part on an estimate, as explained in the methods.

It is not possible to make a comparison with Switzerland on this point due to a lack of official overall figures.

The average number of MPIs per facility increased in all countries (Table 1). It was highest in Switzerland and lowest in Germany. In Switzerland, a shift from MPI SPECT to MPI PET could be observed.

The general increase in the average number of MPI per facility is encouraging, as expertise grows in parallel with the number of studies and serves as an indirect indicator of good quality.

The observation of increasing numbers is consistent with the reported MPI referrals from 2021 to 2024. At least 50% of all facilities in the three countries reported that their MPI referrals had remained unchanged or increased (Austria 50%, Germany 69%, Switzerland 69%). In Switzerland, MRI was cited as the reason for fewer referrals. Germany and Austria cited MRI as well as other procedures, which makes it challenging to identify a single imaging procedure as a competitor. Notably, CT coronary angiography was only listed as a competing procedure in Germany, and there, only in a very few departments. These results are based on perceptions. Reliable figures were not determined.

The referrer structure was roughly the same in all countries. Following the diagnostic decision-making pathway, most referrals came from cardiologists [6]. In Austria and Switzerland, the proportion of referrals from hospitals was clearly higher than in Germany. This was probably due to a shift of hospital examinations to the outpatient sector, which continued to increase during the observation period in Germany.

Stress testing with ergometry continued to play only a minor role. It remained at the very low level of 3% in Austria, at about 40% in Germany, and declined below 10% in Austria. The frequent use of PET MPI explains the strong decline of ergometry in Switzerland. Regadenoson was the most important pharmacological stressor in all countries. In Austria, Adenosine was no longer used by the participating facilities. The slight decline in the use of regadenoson and the corresponding shift to adenosine in Germany was probably due to restrictive reimbursement regulations for regadenoson in some administrative regions.

There were apparent differences between the three countries in the SPECT protocols used. A shift towards two-day protocols was observed in Austria and Switzerland. However, Switzerland continued to have the highest number of SPECT 1-day studies.

Assuming a similar distribution of patients undergoing MPI for CCS in these countries, the resulting proportions of stress-only protocols should have been comparable. Presumably, differences in the diagnostic steps account for the results found (stress-only between 1% Switzerland and 36% Austria).

The camera systems for SPECT MPI were at a high level, with a significant shift towards SPECT-CT systems in Austria and Switzerland. Germany brought up the rear with a predominance of multi-detector cameras and only a slight increase in SPECT-CTs, which was considerably lower than in Austria and Switzerland. CZT cameras recorded a slight increase in all countries, with Austria having the largest proportion of departments.

In line with the camera systems, attenuation correction was performed entirely on a CT basis. The proportion of patients examined with attenuation correction corresponded to the proportion of SPECT-CT examinations in each country. Germany, therefore, had the lowest proportion. One German department still used a transmission source system, which was replaced by SPECT-CT in autumn 2025. It should be emphasized that in Switzerland, all scans were 100% attenuation corrected.

ECG-gated SPECT showed high usage rates (over 90%) in all countries, with the best results seen in Switzerland. The 2021 survey in Austria revealed strikingly low gated rest acquisitions. There have been positive developments in this field over the past three years. The authors would be delighted if their survey project had contributed to this change.

While in 2021, the utilization of perfusion scores still showed differences between the three countries, a significant convergence has occurred since then. The only notable difference is that 10% of departments in Austria do not use it.

Notably, the results of the current survey indicate an ongoing adoption of PET imaging for assessing cardiac disease entities beyond ischemic heart disease, specifically inflammatory cardiopathies and endocarditis. Future surveys will inform the community on the trends in this regard.

## Conclusion

This second joint survey for 2024 on MPI imaging in Austria, Germany, and Switzerland reveals positive developments in MPI imaging in all three countries. Despite differences in procedural issues (such as stress testing and protocols), the results document a high level of guideline conformity.

In terms of Olympic medals, the ranking would be as follows: Switzerland: gold, Austria: silver, and Germany: bronze.

Switzerland’s gold is based on the wide use of PET MPI, camera equipment, and attenuation correction. The German situation reflects country-specific differences in investment priorities and reimbursement structures.

In future, the growing use of CT coronary angiography could lead to a change in the use of MPI imaging in the near future. It is possible that MPI numbers may decrease or even increase—the latter due to inconclusive or intermediate CT results. Future surveys are needed to monitor this development.

## Data Availability

The data on which this article is based are not publicly available in order to protect the privacy of the facilities submitting their data. This was explicitly promised to all participants. Data can be passed on anonymously on reasonable request.
